# Defining the Transcriptional and Cellular Landscape of Type 1 Diabetes in the NOD Mouse

**DOI:** 10.1371/journal.pone.0059701

**Published:** 2013-03-26

**Authors:** Javier A. Carrero, Boris Calderon, Fadi Towfic, Maxim N. Artyomov, Emil R. Unanue

**Affiliations:** 1 Department of Pathology and Immunology, Washington University School of Medicine, St. Louis, Missouri, United States of America; 2 Immuneering Corporation, Cambridge, Massachusetts, United States of America; St. Vincent’s Institute, Australia

## Abstract

Our ability to successfully intervene in disease processes is dependent on definitive diagnosis. In the case of autoimmune disease, this is particularly challenging because progression of disease is lengthy and multifactorial. Here we show the first chronological compendium of transcriptional and cellular signatures of diabetes in the non-obese diabetic mouse. Our data relates the immunological environment of the islets of Langerhans with the transcriptional profile at discrete times. Based on these data, we have parsed diabetes into several discrete phases. First, there is a type I interferon signature that precedes T cell activation. Second, there is synchronous infiltration of all immunological cellular subsets and a period of control. Finally, there is the killing phase of the diabetogenic process that is correlated with an NF-kB signature. Our data provides a framework for future examination of autoimmune diabetes and its disease progression markers.

## Introduction

T1D is a T-cell dependent autoimmunity directed against the β-cells of the pancreatic islets of Langerhans, a process where autoreactive CD4^+^ T cells are directed to antigens of the β-cells[1]–[4]. The most important genetic determinant of T1D incidence in both humans and mice is the major histocompatibility complex (MHC) [5], [6]. Despite the strong genetic component of predisposition to disease, concordance between monozygotic twins is approximately 50% [1], [7]. Beside the interplay of genes and environment in the induction of T1D, there are complex interactions between different immune system cells that determine the outcome of disease [8].

Transcriptional profiling permits an unbiased examination on a genome-wide scale. Thus, it is a powerful tool for analyzing complex problems like T1D. Examination of islets of Langerhans, islet infiltrating leukocytes, and pancreatic lymph nodes has been conducted following several genetic, biological or chemical manipulations[9]–[14]. Several important findings have come from these types of studies. Global expression patterns distinguish destructive versus innocuous inflammatory responses of islet infiltrating leukocytes [9]. Chemically induced diabetes led to upregulation of inflammatory genes with an interferon-γ (IFN-γ) signature in islets of Langerhans [11]. The balance between pancreatic destruction and repair was detected by examining microarrays from T cell-receptor (TCR) transgenic NOD mice [13]. Type I interferon inducible genes were detected in pancreatic lymph node CD4^+^ T cells isolated from TCR transgenic mice [14]. Transfer of diabetogenic T cells from TCR transgenic mice induced an interferon signature that correlated with amplification of the autoimmune process [12]. Finally, the genetic programs that led to development of endocrine pancreas were examined using global transcriptional analysis [10]. Despite these many studies, a coherent view of the disease development and corresponding sequence of inflammatory and developmental events in the context of the spontaneous diabetes is lacking to this date.

A challenge of examining an inflamed islet of Langerhans is the diversity of cell types in the sample and the ability to reliably detect changes in levels of expression. Technological improvements in automation, computation, and reduced cost have allowed an expansion of reliable data gathering, annotation, and distillation using microarray technology. The advent of carefully curated resources such as the Immunological Genome Project provide a framework for identifying genes related to specific cell types [15]. The increase in publicly available and user-friendly resources for microarray data analysis also permits reproducible examination of larger data sets [16]. We have leveraged the power of these available resources to generate a dataset identifying the key signatures of progression of T1D in NOD mice.

Here we present the first chronological examination of the transcriptional landscape of T1D progression in the NOD mouse from 2 wks of age until diabetes (∼20–30 wks). Several striking results were obtained from the dataset. First, infiltrating myeloid cells begin to populate the islets of Langerhans as early as 4 wks of age. This occurred at a time where the islet was still developing, as determined by signatures of cell cycle and development. Next, a type I interferon signature was detected between 4 and 6 wks of age. This is followed by infiltration of islets by all major leukocyte subsets and T cell activation at 8–12 wks of age. Following full immunological activation, there was a progressive enhancement of inflammatory signatures, culminating in the destruction of the islet of Langerhans. This data and its analysis provide a framework for the study of islets of Langerhans and inflammatory responses during the progression of T1D.

## Results

### Examination of Islets of Langerhans Reveals a Progressive Entry of Leukocytes Subsets Throughout Diabetogenesis

**Figure 1:**
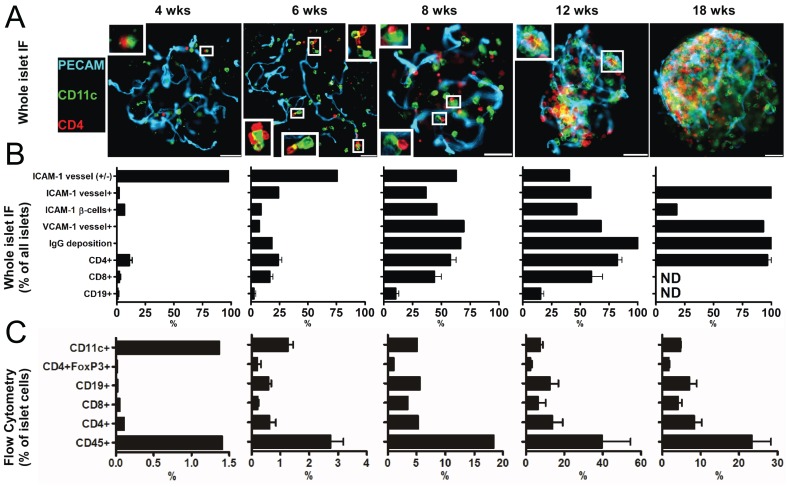
Examination of NOD islets throughout diabetogenesis. (**A**) Islets of Langerhans were isolated from NOD mice at the indicated ages and stained for blood vessels (PECAM-1), intra islet myeloid cells (CD11c), and T cell (CD4). Shown are representative images obtained from a pool of 6 mice per age from two independent experiments. Insets show contacts between intraislet myeloid cells and T cells. White bars represent 50 µm. (**B**) Islets were isolated and stained for the indicated markers and then scored for presence or absence of staining. Bars represent mean+/−S.D. of the percentage of marker positive islets of Langerhans obtained from a pool of 6 mice per group from two independent experiments. (**C**) Islets of Langerhans were dispersed and cells were examined by flow cytometry for the indicated cell surface markers. Bars represent the mean+/−S.D. of the percentage of total islet cells identified in two independent experiments per age. Results were obtained from a pool of 8 to 10 mice per group.

Islets of Langerhans were isolated, purified, and then examined by fluorescence microscopy and flow cytometry at ages ranging from 2 wks up to newly diabetic. Our goal was to identify changes in leukocyte infiltration, changes in adhesion molecules, and immunoglobulin (Ig) deposition on β-cells (Fig. 1).

At 2 wks of age, islets showed no signs of pathology and no detectable lymphocytes. The earliest time point where lymphocytes were identified inside islets occurred at 4 wks of age. CD4^+^ T cells were found in ∼10% of the islets in close apposition to the islet resident CD11c^+^ cells (Fig. 1A–B). The resident CD11c^+^CD45^+^ cells are the intra-islet myeloid cell found in all strains of mice (reviewed in [17]). CD8^+^ T cells and B cells were rarely identified. In control mice, lymphocytes were not detected inside the islets, only an occasional one was found inside the blood vessels (see below for control information). In 4 wk old NOD mice, the islet distribution of the adhesion molecules ICAM-1 and VCAM-1 showed the profile of a non-inflamed state [12]. ICAM-1 was only found weakly expressed in the endothelium and VCAM-1 was absent (Fig. 1B).

In 6 wk old NOD mice, CD4^+^ T cells were found in ∼ 1/3^rd^ of the islets, at a median of 3 cells per islet, and always contacting the islet CD11c^+^ cells (the number ranged from ∼1 to 55 leukocytes per islet; Fig. 1B–C). Low numbers of CD8^+^ T cells also were found while B cells represented a minor component (Fig. 1C). At about this time, many of the intra-islet vessels had higher ICAM-1 expression throughout most of the vessel wall, and about 10% showed VCAM-1 expression (Fig. 1B). These changes in adhesion molecules have been related to the entrance of diabetogenic T cells [12], [18]. IgG deposition was found on the β-cells in many of the islets (Fig. 1B). The islets of normal mice never showed deposition of IgG suggesting the presence of NOD-specific autoantibodies.

Major islet changes were found at 8 wks of age. The total number of leukocytes rose ∼7-fold, from ∼2.7% to 18% of the total islet cellularity (Fig. 1C). CD4^+^ T cells and CD8^+^ T cells were found in about half of the islets but B cells only in a limited number, about 10% (Fig. 1B). There was also an increase in the number of CD11c^+^ cells at this stage (Fig. 1C). We found increased expression of ICAM-1, even on the β-cells, VCAM-1 expression on the vessels, and IgG deposition on the β-cells (Fig. 1B). At 12 wks of age, peri-insulitis, a leukocytic lesion on the pole of the islets, was evident in some islets (Fig. 1A). By 18 wks, islets were heavily infiltrated making quantification difficult (Fig. 1).

Thus, between 4–6 wks the islets of Langerhans showed the first signs of pathology with the entrance of T cells and signs of islet reactivity. Starting at ∼8 wks there was a progressive infiltration of all major leukocyte subsets and signs of inflammatory marker expression. The same biological replicates that were interrogated by microscopy were analyzed for their transcriptional expression profiles as detailed below.

### Assembling the Compendium of Transcriptional Profiles in the Context of Islet/Diabetes Development

To perform chronological analysis of the transcriptional profile, the NOD strain was compared with several control strains. Islet RNA was extracted from 3–6 replicates for each of seven time points along the lifetime of NOD mice (2, 4, 6, 8, 12, 18 wks and up to diabetics at ∼20 wks) and compared to 6 replicates of 3 control strains. NOD.*Rag1^−/−^* mice at 2 and 6 wks of age were used as a control because they lack the adaptive arm of the immune response, but are otherwise genetically identical to NOD, allowing us to trace non-immune developmental processes in islets. Two additional controls strains were used in this study to increase the robustness of the analysis. The B6.NOD-H2*^g7^* strain (B6.g7) was chosen because it expresses the MHC-II protein I-A^g7^ on a C57BL/6 background. This strain does not develop diabetes. We also used C57BL/6 mice as our most normal control strain.

Examination of the entire data set by Spearman’s rank correlation showed a low variance amongst all data (Fig. S1A, Spearman’s Rank <0.089). By Spearman’s Rank, the data was grouped mainly by the age of the mice. Similarity in variance amongst the 2 and 6 wk old mice was independent of background strain. The outliers in the data set were the 8–18 wk NOD mice and the newly diabetics. In particular the 8 wk NOD mice were most distant in variance from the rest of the data.

### The Earliest Transcriptional Difference between NOD and Control Mice, Including NOD.Rag1^−/−^ Mice, was an Interferon Signature

**Figure 2:**
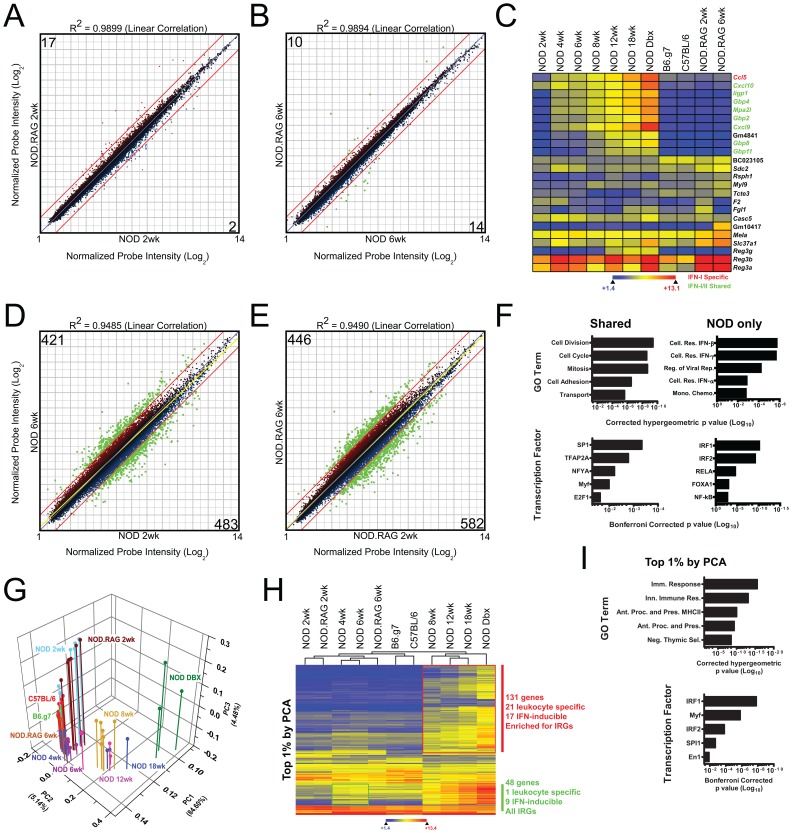
Pairwise and principal component analysis of microarray data. (**A–B**) Scatter-plots of the normalized probe intensity of all annotated microarray signals are shown. Each dot represents the mean of 6 independent biological replicates. Numbers in the box represent the number of features that were at least 2-fold different at a 99% confidence interval by moderated t test with Benjamini-Hochberg false discovery rate analysis. Data are plotted at a log_2_ scale. Panel (**A**) compares NOD.*Rag^−/−^* mice versus NOD mice at 2 wks of age and panel (**B**) compares them at 6 wks of age. (**C**) Hierarchically clustered heat map (Euclidean row distance) of the 24 differentially expressed transcripts identified in (**B**). (**D–E**) Scatter-plots as in (**A–B**) except the comparison is between 2 and 6 wk old NOD or 2 and 6 wk NOD.*Rag^−/−^*. (**F**) GO terms and transcription factor binding analysis of the differences identified in (**D–E**). The two graphs on the left, labeled ‘Shared’ show signatures common to both NOD and NOD.*Rag^−/−^*. The signatures on the right, labeled ‘NOD only’, show changes specific to the NOD strain. (**G**) Principal component analysis of microarray samples. Each of 57 microarrays is summarized as a point and drop-line. Samples are color coded as indicated by the title of each group. (**H**) Hierarchical clustered heat map (Euclidean distance) of the top 1% most variant genes identified by principal component. (**i**) GO and transcription binding analysis of the top 1% most variant genes identified by principal component analysis.

First, we looked at transcriptional difference between the two genetically closest diabetic and non-diabetic strains (NOD and NOD.*Rag1^−/−^*). Since the main difference between these mice is the presence of an adaptive immune response, we reasoned that only immune system dependent changes would be found between the two strains. We performed pair-wise comparisons of NOD and NOD.*Rag1^−/−^* mice at 2 and 6 wks of age (Fig. 2A–F). At 2 wks of age, there were 19 detectable differences between NOD and NOD.*Rag1^−/−^* mice. Overall, there were very few differences between 2 wk old NOD and NOD.*Rag1^−/−^* mice and no obvious differences related to immune responses.

By 6 wks of age, we found 24 significant differences between NOD and NOD.*Rag1^−/−^* (Fig. 2B–C). We found 14 genes more highly expressed in NOD than NOD.*Rag1^−/−^* and 10 genes more highly expressed in NOD.*Rag1^−/−^* than NOD. The transcripts with higher expression in NOD were all IFN-inducible transcripts. For our study, interferon-inducible genes were defined based on the microarray analysis of Liu, et al. [19]. The interferon signature increased in magnitude over the time course of NOD development, and was not found in any of the other control non-diabetic mice (Fig. 2C). (Many of the expression differences between 6 wk old NOD and NOD.*Rag1^−/−^* mice were detectable as early as 4 wks of age in NOD; see below for details.).

Next we interrogated changes that took place between 2 and 6 wk old mice in NOD or NOD.*Rag1^−/−^* mice. There were ∼1000 significant changes between 2 wk and 6 wk old NOD and between 2 wk and 6 wk old NOD.*Rag1^−/−^* mice (Fig. 2D–E). Gene ontology and promoter scanning of the genes commonly upregulated in both strains showed that they were involved in cell division, cell cycle, and cellular adhesion. They also included promoter binding sites for early developmental regulators, such as SP1 (Fig. 2F). This indicated that the islet of Langerhans was still actively dividing and developing from 2 to 6 wks of age regardless of diabetes susceptibility.

In addition to upregulated genes, a number of transcripts were downregulated between 2 and 6 wk NOD and NOD.*Rag1^−/−^* mice. The level of downregulated transcripts was comparable in all strains, including B6.g7 and C57BL/6 mice. Analysis of variance of the 6 wk downregulated genes by Spearman’s rank grouped most of the 6 wk samples together, and the main outlier was the 8 wk NOD (Fig. S1B). Analysis of the downregulated signature showed an enrichment of transcriptional regulatory genes (Fig. S1C). Euclidean distance and Pearson’s Correlation analysis did not provide a discernible trend in the downregulation of transcripts through the time course of NOD mice development (Fig. S1D, Data not shown).

In summary, pairwise analysis of 2 and 6 wk old NOD and NOD.*Rag1^−/−^* mice showed mostly a regulation of cell growth and development, and an inflammatory response unique to NOD. A surprising finding was the fact that as early as 6 wks of age an interferon-dependent gene signature was already found in NOD mice, but not in any of the 6 wk old controls. Many of the 6 wk inflammatory changes were evident in NOD mice as young as 4 wks, as will be detailed below.

### Principal Component Analysis Revealed that the Major Source of Transcriptional Variance between NOD and Control Mice was Immunological in Nature

Principal component analysis (PCA) was used to evaluate the transcriptional changes responsible for the highest variance of the dataset [20]. PCA discriminated our samples by both strain and age (Fig. 2G). We extracted the top 1% of transcripts responsible for the overall PCA variance and this yielded transcripts that correlated with a progression in the age of NOD mice (Fig. S1C). Two salient features were identified by PCA and noted in Fig. 2H. The first was a group of genes selectively upregulated as early as 4 wks of age in NOD mice. These were immune response genes, including the interferon inducible genes mentioned in Fig. 2C. A second group of genes was selectively upregulated on or after 8 wks in NOD mice. This group was enriched for immune response and a variety of leukocyte specific and interferon-inducible genes. Key leukocyte cell surface markers, including *Cd2*, *Cd3e/d/g*, *Cd4*, *Cd8a*, *Cd19*, *Cd22*, T cell receptor, B cell receptor and immunologically important signaling components such as *Lck*, *Lat*, and *Cd79b*, were identified by PCA. Analysis by Gene Ontology and promoter scanning showed that genes identified by PCA were significantly enriched for immune response and antigen processing and presentation signatures and IRF1 was the most significant transcriptional regulator (Fig. 2I).

### Refined Staging of NOD-specific Transcriptional Changes in the Islets of Langerhans

In order to better identify transcriptional changes positively correlated with a progression toward diabetes, we performed k-means clustering using uncentered Pearson’s correlation. Our goal was to focus on genes with a positive Pearson’s correlation throughout diabetogenesis and with lower relative expression in all our controls.

The dataset was clustered as shown in Fig. S2A. Several clusters contained groupings of genes with a positive correlation of expression throughout diabetogenesis. The clusters that were chosen were highlighted in red and demonstrated an increase in gene expression intensity throughout diabetogenesis, but had relatively lower expression levels in all controls. Using this analysis we identified 888 genes that were selected for further analysis. Pearson’s correlation excluded most of the developmental changes mentioned previously, but included immune-related genes changes missed by pair-wise analysis and PCA (Fig. S2C). Also, Spearman’s rank correlation analysis of the genes identified by Pearson’s showed a delineation of NOD mice at different ages (Fig. S2B).

**Figure 3:**
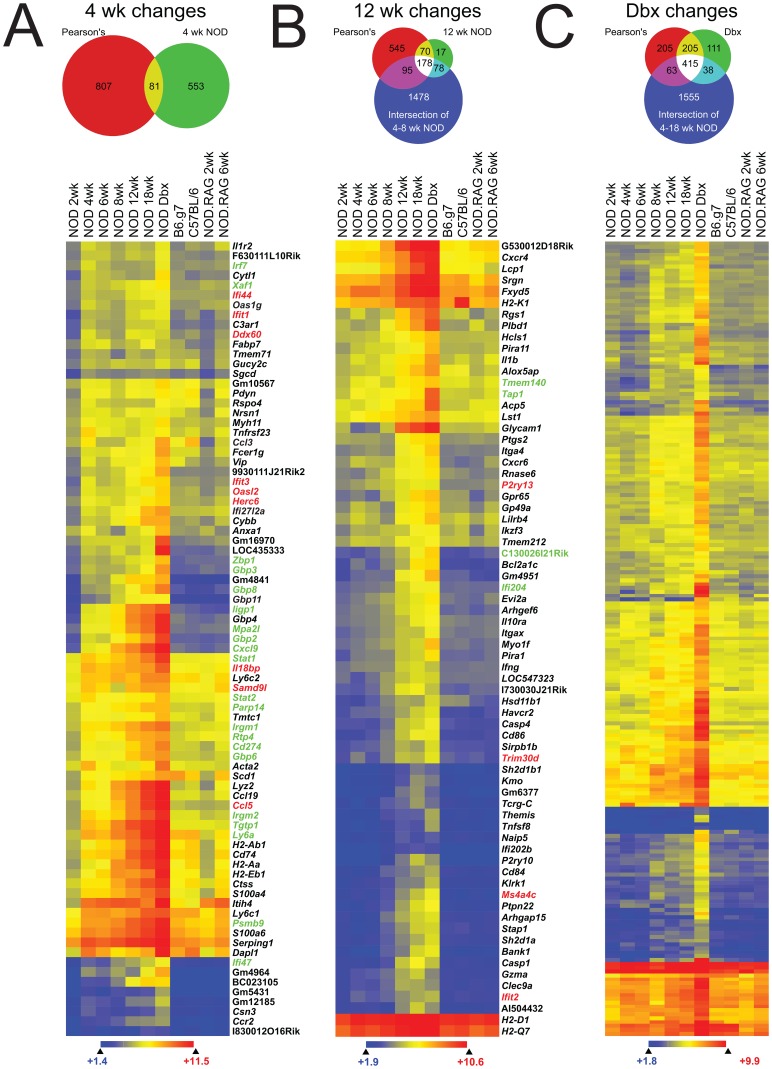
Identification of significant gene changes in different aged NOD mice. Examination of transcriptional changes that took place between 2 wks and either (**A**) 4 wks, (**B**) 12 wks, or (**C**) newly diabetic NOD mice. The red portion of each Venn diagram shows the genes identified by Pearson’s correlation as following a positive correlation throughout diabetogenesis. The green portion of each Venn diagram shows statistically significant changes from 2 wks to the given wk as determined by 2-fold upregulation and 99% confidence interval by moderated t test with Benjamini-Hochberg false discovery rate analysis. The blue portion of the Venn diagrams is the intersection of all the pairwise statistically significant differences from 2 wks to the indicated ages. Hierarchically clustered heat maps show the Euclidean distance of genes identified by the yellow intersection in the Venn diagrams. Those were the genes that showed both a positive correlation by Pearson’s correlation and a pairwise fold and statistical change at the indicated time. Gene names in red are type I interferon-inducible and those in green are inducible by both type I and type II interferons.

The genes identified by Pearson’s were superimposed with pair-wise comparisons performed between 2 wk old NOD and the age in question. This allowed us to establish genes that were significantly upregulated in NOD mice of different ages. For example, there were 634 significantly upregulated genes between 2 wk and 4 wk old NOD mice (2-fold, 99% C.I.) and the intersection of these changes with Pearson’s Correlation yielded 81 significant changes at 4 wks (Fig. 3A, Table S1). These 81 genes included all 14 of the upregulated genes identified in Fig. 1A–C. The remaining genes were upregulated ≥2-fold in NOD and to a lesser extent in the controls (see below).

For subsequent time points, we combined the intersection of Pearson’s Correlation with pairwise statistics at a given age (6, 8, 12, 18, and newly diabetic), but excluded changes already significant at a previous time. Fig. 3B–C shows an example of this for 12 wk and newly diabetic changes. In the case of the 12 wk sample, 70 new significant changes were detected and in newly diabetic mice 205 new significant changes were detected. We used the changes identified this way to determine the presence of immune signatures during diabetogenesis.

### Detailed Analysis of Immune Signatures during T1D

**Figure 4:**
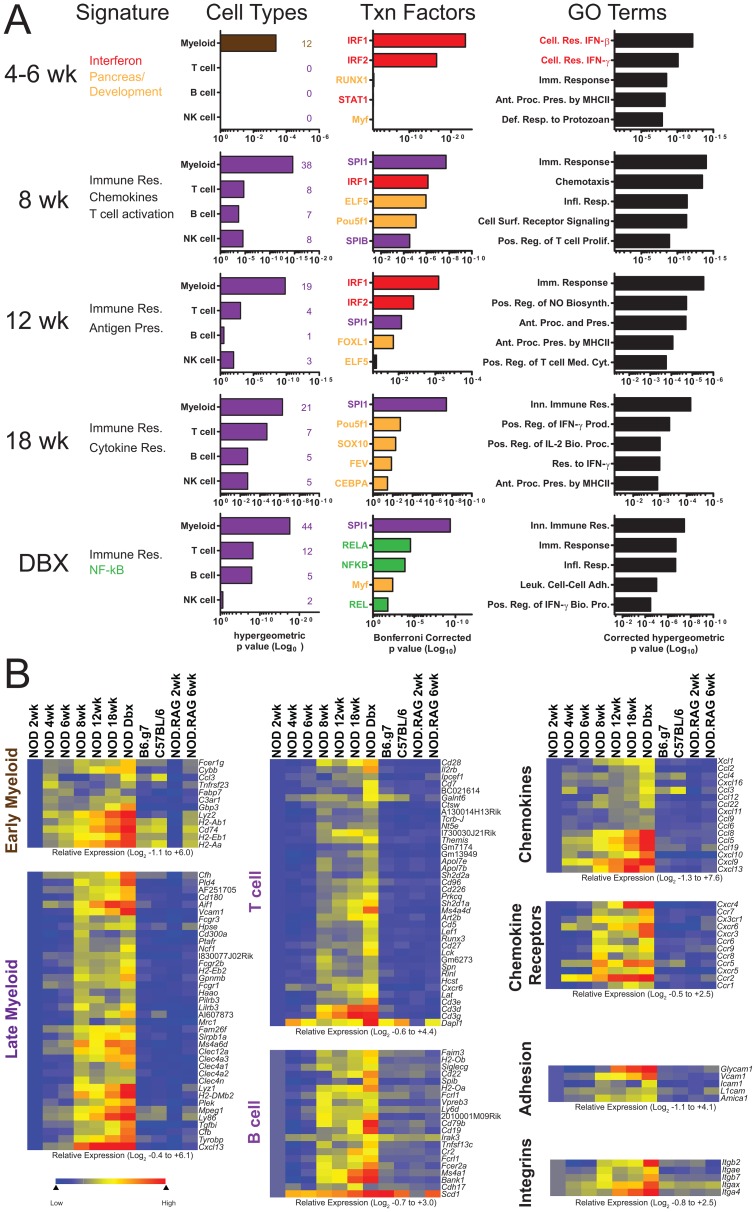
Analysis of inflammatory genes changes throughout diabetogenesis. Differentially expressed genes identified in Figure 3 and Supplemental Table 1 were interrogated for transcriptional regulation signatures and gene ontology (**A**) or for their immunological role (**B**). (**A**) Cell enrichment, transcription factor binding, and gene ontology analysis were performed. Numbers in parentheses indicate the number of cell type specific genes identified as statistically significantly changed at the given age. (**B**) Hierarchically clustered heat maps of cell type specific gene changes throughout diabetes. Values were adjusted to a per row color scale so all changes were relative to 2 wk NOD mice.

To dissect the transcriptional profiles of T1D progression, we combined transcription factor binding analysis (p-scan), gene ontology, and the modules and regulators dataset of the Immunogenetics Consortium (Immgen) [15], [21]–[24]. The idea was to add power to our approach by using groups of genes instead of analyzing any single individual transcript. Based on this approach, we parsed out the results previously shown in Fig. 3 and Table S1 as shown in Fig. 4.

At 4–6 wks of age NOD mice displayed an interferon transcriptional signature, determined by the enrichment of IRF1 and IRF2 binding sites on the promoters of upregulated genes. Cellular response to IFN-β and IFN-γ were the two most enriched GO Terms. There was also a significant but weak developmental signature (RUNX1 and Myf; Fig. 4).

Using Immgen’s modules and regulators we found 12 genes that were myeloid specific and that we termed as an “early myeloid” signature (Fig. 4B). Most of the early myeloid signature was found in all strains, but increased over time in NOD mice. The early myeloid genes were not specific for any identifiable leukocyte, but many were linked to antigen processing and presentation (*H2-Ab1*, *H2-Eb1*, *H2-Aa*, and *Cd74*). This signature is compatible with an intra-islet myeloid cell previously described by our group and others [25]–[27].

By 8 wks of age, there was a significant upregulation in genes regulated by SPI1 (myeloid regulator) and SPIB (B lymphocyte regulator). At this time, all major leukocyte subsets implicated in the development of diabetes were represented transcriptionally. Gene ontology showed enrichment of chemotaxis and T cell activation signatures. Definitive T cell specific genes including *Cd2*, *Cd3d*, *Cd3g* and also a signature of T cell activation as defined by *Ctla4*, *Slamf1*, *Slamf6*, *Slamf7*, *Slfn1* (Shlafen1), and *Cxcr5* was defined. B cell specific genes, including the immunoglobulin and Ig-associated genes *Igk*, *Igl*, and *Cd79b* were also detected (Fig. 4, Table S1). Natural killer cells were identified by the induction of genes belonging to the NK gene cluster (*Klrc1, Klrd1,* and *Klra17*; Table S1) [28]. In 8 wk old NOD, we found 38 new myeloid transcripts, which we termed the “late myeloid” and included markers enriched on monocytes, macrophages, and dendritic cells, such as 5 members of the c-type lectin family *Clec4a1*, *Clec4a2*, *Clec4a3*, *Clec4n*, *Clec12a* (Fig. 4B and Table S1).

At 12 wks of age a second wave of IRF1/IRF2 response was detected. This corresponded to a GO profile that included antigen processing and presentation and positive regulation of T cell mediated cytotoxicity (Fig. 4A). At 18 wks there was further amplification of the response, increased IFNγ and IL-2 dependent gene expression, and a continued myeloid signature as evidenced by SPI1 regulated transcripts. Finally, newly diabetic mice showed an enrichment of NF-κB inducible transcripts (Fig. 4A).

Examination of transcriptional changes by Pearson’s correlation and ANOVA demonstrated that the inflammatory gene upregulation during diabetogenesis occurred in 5 major patterns. Coordinated upregulation of various transcriptional groups was found at 4, 8, and 18 wks as well as in newly diabetic mice. There was also a steady upregulation profile (Fig. S3).

Chemokines are considered the drivers of specific immune cell entry into tissues. We found 4 chemokine genes were upregulated by 4 to 6 wks: *Ccl3*, *Ccl5*, *Ccl19* and *Cxcl9* (Fig. 4B). Of these, *Ccl5* and *Cxcl9* were higher in NOD than in controls. None of the chemokine receptors for *Ccl5* or *Cxcl9* were detectable in 4 or 6 wks old NOD. The only chemokine receptor to be found significantly upregulated in NOD at 4 and 6 wks was *Ccr2*, a receptor enriched in blood monocytes but also expressed on some populations of dendritic cells. Therefore, at 4 and 6 wks there was not a detectable chemokine-chemokine receptor pairing that could be responsible for the entry of any specific leukocyte subset. However, by 8 wks of age there was NOD-specific upregulation of 11 chemokines and 7 chemokine receptors (Fig. 4B). The chemokines included ligands for recruitment of naïve (*Ccl19*), memory (*Cxcl9/10*), and activated αβ T cells (*Cxcl9/10*). We also detected at least one ligand for the recruitment of γδ T cells (*Ccl22*), regulatory T cells (*Ccl22*), B cells (*Cxcl13*), NK cells (*Ccl4*, *Cxcl11*), conventional and plasmacytoid DC (*Cxcl11*, *Ccl4*), blood monocytes (*Ccl2*), macrophages (*Ccl2*, *Ccl5*), and neutrophils (*Xcl1*). Chemokine receptors for all major leukocyte populations were also found, with the exception of granulocytes. The pattern of chemokine and chemokine receptors remained mostly unchanged after 8 wks of age, except for the increase of *Cxcr4* and *Cxcr6* at 12–18 wks of age. The data show that at the chemokine/chemokine receptor expression level the ability to recruit all major leukocyte subsets involved in the progression of diabetes was in place by 8 wks of age.

Our approach detected 5 significantly upregulated adhesion molecules: *Glycam1*, *Vcam1*, *Icam1*, *L1cam*, and *Amica1*. All adhesion factors were significantly upregulated only in the NOD mice. *Vcam*, *Icam1* and *L1cam* were upregulated by 4 to 6 wks of age, *Amica1* was upregulated by 8 wks of age and *Glycam1* was upregulated by 12 wks of age. *Glycam1* was the most upregulated adhesion factor throughout diabetogenesis. Integrins that were significantly upregulated after 12 wks of age included *Itgb2* (CD18), *Itgae* (CD103), *Itgax* (CD11c), *Itga4* (CD49d), and *Itgb7*. Overall the adhesion factor and integrin signature points to an enrichment of myeloid cell types. A concomitant upregulation of expression of the pan-leukocyte marker *Prptc* (CD45) and modest changes in islet-specific genes were also detected (Fig. S4).

### Examination of Interferon and Interferon-inducible Gene Expression by Quantitative RT-PCR

**Figure 5:**
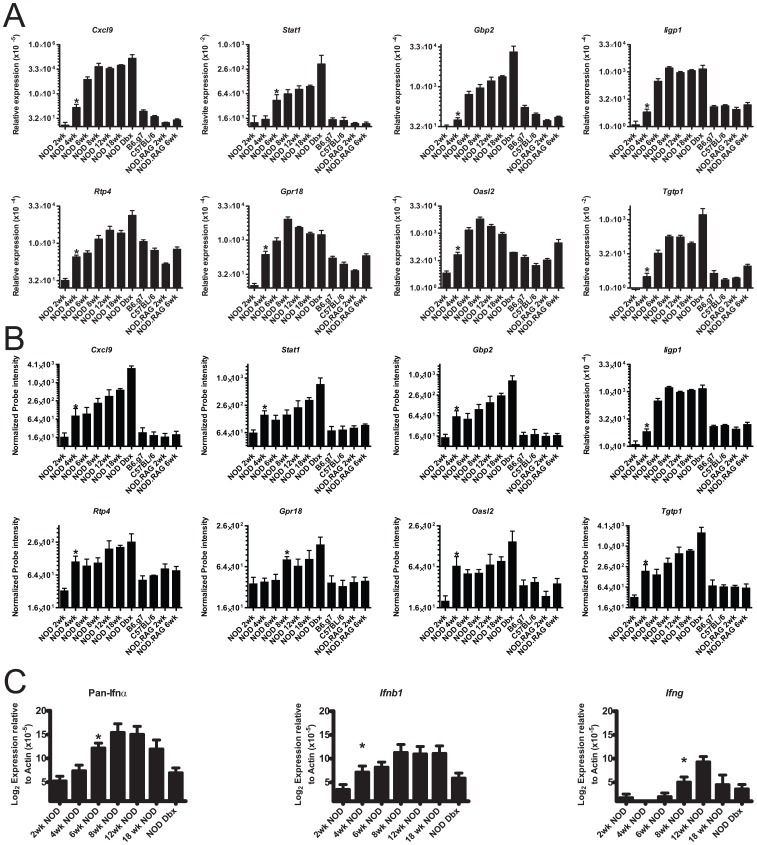
Quantitative RT-PCR validation of microarray data. (**A**) Quantitative RT-PCR was performed using SYBR green detection for the indicated genes. Bars show the mean (log_2_) +/− S.E.M. of at least three independent experimental replicates from 3–6 biological replicates per group. All data is represented relative to the expression of actin (ΔC_t_). In order to facilitate visualization on a log_2_ scale, values were transformed as indicated on the y-axis label. (**B**) Microarray results for the same genes interrogated in (**A**). (**C**) Taqman qPCR quantification of pan-IFNα, *Ifnb1*, or *Ifng* throughout diabetogenesis. Bars represent the mean of the normalized probe intensity +/− S.E.M. of 3–6 biological replicates per group. Asterisks indicate statistical significance (P<0.05) from 2 wk NOD sample by one-tailed Mann-Whitney test.

We validated 8 candidates genes that were significantly upregulated between 4 and 8 wks in our microarray data set using qRT-PCR: *Cxcl9*, *Stat1*, *Gbp2*, *Iigp1*, *Rtp4*, *Gpr18*, *Oasl2* and *Tgtp1*. Fig. 5 shows the results of the RT-PCR data (Panel A) and microarray data (Panel B) for the 8 validated targets. By RT-PCR there was significant upregulation of all 8 transcripts in 4 to 6 wk old NOD mice when compared to 2 wks. *Cxcl9* and *Gbp2* were significantly upregulated from 2 to 4 wks in NOD and were significantly different from all control strains at 6 wks of age (Fig. 5). As with our microarray results, certain genes, like *Rtp4* were upregulated in NOD from 2 to 4 wks, but had comparable expression levels with controls at 6 wks of age. Both of these observations were in agreement with the microarray results and support the idea that there is an entry of leukocytes that is universal to all strains of mice and a second signature that is interferon-inducible enriched and specific to NOD (Fig. 5B, Table S1).

We wanted to determine which interferons were transcribed in the islets of Langerhans. We reliably found a “tonic” transcript for *Ifna* in the islets, pancreatic lymph nodes and spleens of all strains of mice and at all times examined (Fig. 5C which only shows data from islets). There was a small level of upregulation from 2 to 4 wks in NOD mice, but expression levels were 10 to 100-fold higher than in the three control strains. *Ifnb* transcripts also were reliably detectable in the islets of all mice, and sporadically in the spleens and lymph nodes of NOD (Fig. 5C, data not shown). The levels of *Ifnb* expression did not go above the tonic expression level until 6 wk of age. At this time there were >10 fold more detectable transcripts in NOD than in C57BL/6, B6.g7 or NOD.*Rag1^−/−^* mice islets. To note, pancreatic lymph nodes of NOD mice contain a detectable expression of type I interferon in plasmacytoid DC, as early as 3 wks of age [14].

In contrast to type I interferon, we did not detect tonic expression of *Ifng* in the spleens or lymph nodes of NOD or the control mice (Fig. 5C, and data not shown). Reliable expression of *Ifng* was only detected at 8 wks with maximal expression at 12 wks in NOD. This coincided with the first detectable *Ifng* by microarray. Based on this data, we conclude that type I interferon was expressed before *Ifng* during diabetogenesis. The timing of significant upregulation of *Ifna* and *Ifnb* appears to be simultaneous.

### Integration with Protein-protein Interaction Networks Shows Immune Subnetworks Dominating Across Diabetes Development

We related the islet gene expression data in the context of established protein-protein interaction networks. First, we dissected the temporal changes in gene expression of our dataset by computing pairwise p values for all permutations of samples. This was done in order to identify the genes that became involved in diabetogenesis at a given age. For example, at 8 wks of age, we looked for differential expression from 2 to 6 wk NOD versus 8 to newly diabetic NOD. By this analysis, only genes that became significantly upregulated at 8 wks, but were not significant at all previous times were analyzed.

**Figure 6:**
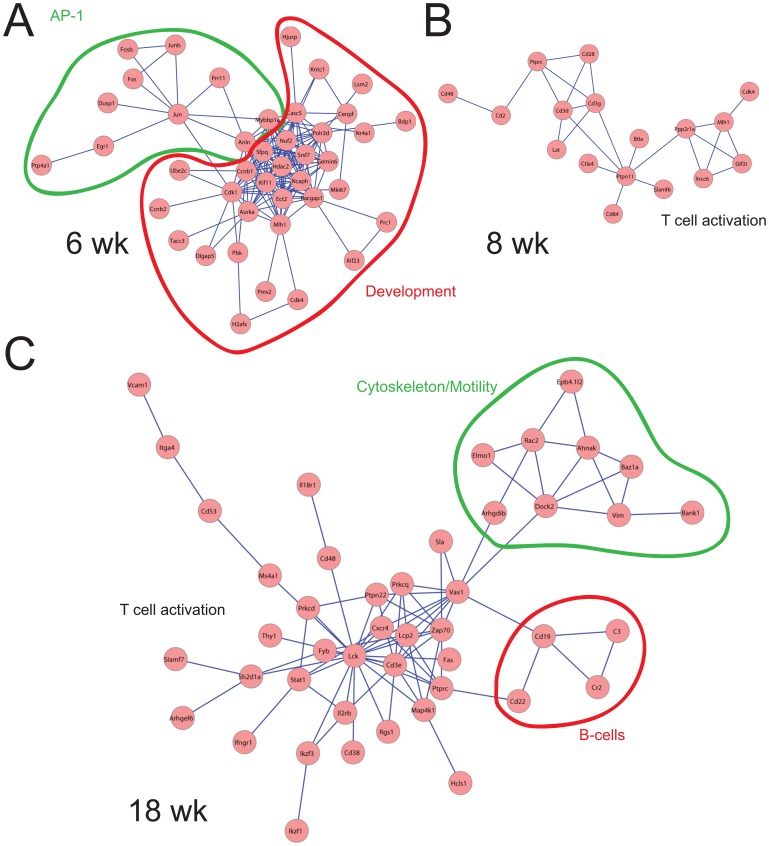
Data modeling of transcriptional networks. The most significant interaction networks were calculated at (**A**) 6 wks (FDR 0.00006), (**B**) 8 wks (FDR = 0.0000001) and (**C**) 18 wks of age (FDR = 0.00012). Interaction significance was based on the p values calculated for each age group. Notably, at 6-wk, strong developmental signature persists along with AP-1 module, generally attributed to myeloid cells. At 8-wks, strongest interacting subnetwork is T-cell specific, while 18-wk additional B-cell and cytoskeleton specific modules appear.

Subnetworks of genes upregulated at 6 wks contained a cluster of AP-1 related immune genes specific for myeloid lineage (*Fos*, *Junb*, *Jun*, *Fosb*, etc). The 8 wk subnetwork was strongly enriched for “Genes involved in Signaling in Immune system” (Reactome, hypergeometric p value <10^−12^) and “T Cytotoxic Cell Surface Molecules” (Biocarta, hypergeometric p value <10^−10^). The 18-wks subnetwork showed strong enrichment in members of both T-cell and B-cell receptor pathway (hypergeometric p values of 10^−6^ and 10^−5^, respectively; Fig. 6).

We expanded these observations by cross-referencing time point specific genes with profiles available in the Immgen database [15]. Since Immgen-derived transcriptional modules available online are defined based on the human immune cell types, we used the enrichment score strategy introduced in Benita, et. al. to compute the representation of murine immune cell types within transcriptional signatures of infiltration [29]. Consistently, the 4 wk NOD signature did not show statistically significant enrichment in immune cell types, while the 6 wk NOD signature showed non-cell type specific increase of immune cell types illustrated by hematopoietic progenitor transcriptional signatures. Strikingly, the 8, 12, 18 wk, and newly diabetic NOD mice showed a specific sequence of immune infiltration. In 8 wk NOD mice transcripts infiltration by CD8 and γδT cells was enriched (Fig. 6 and Fig. S5). This was followed by a myeloid cell re-infiltration at 12 wks, and B cell infiltration at 18 wks. Finally, newly diabetic NOD mice had strong cytotoxic helper T cell signature. This was consistent with both direct observation of islet-infiltrating cell-types and strongest interacting subnetworks identified in the data (Fig. 6), and showed that expression profiles were informative for dissecting heterogeneous cell populations.

## Discussion

Several important milestones in the development of diabetes were derived from a chronological transcriptional analysis that was complemented by cytological analysis of the islets. Examination of individual genes can introduce observational bias. However the addition of Immgen, promoter scanning, ontology analysis, and modeling added an important layer of additional information. This approach allowed us to parse complex lists of genes and cells into an improved definition of the steps leading to T1D and to identify different stages in its progression. Previous microarray analyses of T1D used either accelerated methods for inducing disease or interrogated a subset of the pancreatic infiltrate[9], [11]–[13], [30]. Thus, the events leading to diabetes were temporally compressed or only a subset of the response was analyzed. Despite these limitations, important candidate markers for diabetes were identified, including genes that distinguish progressive versus non-progressive disease [30]. We have greatly expanded on our available datasets and present the first complete analysis of the entire natural diabetic program.

The time at which NOD mice become diabetic is heterogeneous and gender dependent. Despite the stochastic endpoint, the progression of the program that culminates in overt disease was relatively conserved and several important milestones were identified. During the first 4 wks of life, myeloid cells populated the islets, independently of diabetes susceptibility. The first major transcriptional event that differentiated the NOD from control mice was the expression of type I interferon, reliably detected by the 4^th^ wk of age. The expression of type I interferon was correlated with a discrete appearance of CD4^+^ T cells inside the islets. The second milestone was the coordinated infiltration of islets by all major inflammatory cells by the 8^th^ wk of life. This event was synchronous with markers of T cell activation and a dramatic change in islet-specific gene expression. Between the 12 and 18^th^ wk there was further diversity in the expression of genes linked to cytotoxicity, many of which were NF-κB inducible. Despite a prolonged inflammatory infiltrate starting at 8 wks, mice did not become diabetic until after 20 wks of age, indicating a partial level of control for a period of time.

The earliest expression of the autoimmune state was the type I interferon signature, its importance cannot be underestimated. It was not unexpected to find a level of constitutive type I interferon in the islets as well as in other tissues [31], [32]. The key event however was the marked upregulation at 4 to 6 wks of age. Future experiments will need to address the causes and effects of the type I IFN expression. For one, we need to explain the relationship between type I IFN production and the finding of early infiltrating T cells, the specificity of which needs to be examined. Because of their striking contact with islet APC these T cells should be recognizing β-cell antigens. Surprisingly, genes linked to T cell activation were not detectable until 8 to 12 wks of age in NOD. The type I interferon induced *Cxcl9* that develops early in NOD has the ability to recruit both pathogenic and regulatory T cells that bear the *Cxcr3*, the receptor for *Cxcl9*. It is possible that the naïve T cell is incapable of being diabetogenic until it has been converted to an effector/memory phenotype at distal sites, such as the pancreatic lymph node, a site which has been shown important in diabetes development [33], [34]. T cells that initiate the diabetic process may become regulated at 8 wks, but the entry of effector memory cells that are conditioned in the lymph node may cause the process to move forward starting at ∼12 wks.

Concerning other stimuli, we have no evidence for viral infections in our colony, an issue that has been examined with care. But pointedly, gnotobiotic NOD mice still developed diabetes, although the microbiome did influence its development [35]. Nucleic acids have been defined as the most potent agonists of type I interferon production [36]. DNA released by apoptotic β-cells could activate nucleic acid sensor pathways for the induction of type I interferon. Inflammatory nucleic acids may result from the developmental changes that were detected during the first 4 wks. Importantly, an early apoptotic event has been discussed as a possible inductive phase of diabetes [37]. Alternatively, multiple signals may be required for the type I interferon burst which coincides with the initiation of immune infiltration. This hypothesis has been recently postulated for the induction of lupus, a type I interferon mediated autoimmunity [38]. Careful evaluation of different nucleic acid sensor pathways in NOD is lacking and is an important future direction.

Finally, we note some of the reports in the literature that reinforce the importance of a type I interferon response. Analysis of RNA isolated from whole pancreatic lymph nodes demonstrated an upregulation of type I IFN in NOD mice from 2 to 3 wks [14]. However, it is not clear if this upregulation is unique to NOD mice. Treatment of mice with type I interferon neutralizing antibodies delayed disease incidence. Higher level of *Ifna* expression was found in the pancreases of human T1D patients with end-stage disease when compared to normal controls [39]. Diabetes prone BB rats and mice treated with streptozotocin had expression of type I interferon in their pancreatic islets [40]. There are reports of patients treated with type I interferon for chronic viral infections that then developed diabetes [41]. In the case of mice, enforced expression of type I interferon in islets caused autoimmune diabetes [42], [43]. The transcription factor *Irf7*, a key regulator of type I interferon upregulation, has been linked to T1D by genome-wide association studies [44]. There is an abundance of data demonstrating the importance of type I interferon in other autoimmune diseases [45].

The microarrays at 8 wks of age not only marked the detection of all major leukocyte subsets, but also showed changes in islet specific gene expression. We detected downregulation of transcription factors essential for islet development and function. Additionally, by variance analysis these arrays were more distant from all other arrays regardless of whether the genes were unfiltered, selected by PCA, or by Pearson’s correlation. We believe, as do others, that this is due to a control mechanism established in the islets that must be overcome before disease can progress [30]. Multiple genes involved in T cell regulation such as *Ctla4*, *Cd52*, *Havcr2*, *Cd244*, *Btla*, *Cd200*, and *Cd274* were upregulated at 8 wks of age, indicating the potential presence of a control mechanism. By microscopy we were able to detect FoxP3+ regulatory T cells in the islets of NOD mice at 8 wks of age. The demonstration of the importance of regulation in control of disease was shown in NOD mice deficient in functional FoxP3, which developed diabetes at a faster rate than wild-type control mice [46], [47]. The concept of a regulatory balance at ∼8 wks has been shown by altering Treg:Teff levels using IL-2 treatment [48]. Removal of Foxp3+ Tregs in a BDC2.5 TCR transgenic model of T1D revealed an increase in *Ifng* signatures, which was associated with progression of disease [49].

In addition to T cell/Treg specific signatures, examination of NOD mice showed that macrophage gene expression profiles, in particular the expression of *Vsig4* (CRig), was predictive of progression to disease [30]. The presence of a CRig+ macrophage at 10 wks was indicative of a resistance phenotype, and modulation of this population by CRig-Fc treatment protected NOD mice from becoming diabetic. It will be interesting to determine if the inflammatory changes detected between 8 and 12 wks are related to the loss of the protective macrophage signature and whether this is the final trigger that leads to overt diabetes.

Concerning *Ifng*, we found expression at 8 wks in NOD mice, but the maximal signal was not detected until 12 wks of age. Signaling induced by this cytokine results in different biological effects depending on the cellular targets and their activation state. In NOD protective as well as proinflammatory responses have been reported [50], [51]. Whether *Ifng* expression in early versus late diabetogenesis results in different biological responses remains an issue for future consideration.

In sum, discrete transcriptional profiles were identified in the diabetic autoimmunity of the NOD mouse, a chronic progressive and multifactorial disease. This information may set the base to analysis and identification of causative events but also to better define gene markers that can be used to identify the stages of the human disease.

## Materials and Methods

### Mice

NOD (NOD/ShiLtJ), NOD.*Rag1^−/−^* (NOD.129S7(B6)-Rag1^tm1Mom^/J) and B6.g7 *(*B6.NOD*-*H2^g7^) mouse strains were bred at Washington University School of Medicine. C57BL/6 mice were obtained from The Jackson Laboratory (Bar Harbor, ME). All of the mice used in this study were female mice. We identified the diabetic mice following two consecutive daily blood glucose readings ≥250 mg/dl. In our NOD colony, the incidence of diabetes in female mice is ∼90% with incidence ranging between ∼20–30 wks of age. All of the 18 wk old mice had normal blood glucose for at least two days before being sampled. All mouse experiments were approved by the Division of Comparative Medicine of Washington University School of Medicine (Association for Assessment & Accreditation of Laboratory Animal Care (AAALAC) accreditation number A3381-01). Experiments were performed under institutional guidelines and all efforts were made to minimize suffering. The institutional approval number for these studies was protocol number 20110150.

### Islet Isolation and Handling for Immunofluorescence

Islets were isolated with some modifications of the original protocol [52]–[54]. Briefly, pancreata were isolated and treated with collagenase, followed by several steps of centrifugation and washing, and finally, islets were hand-picked. Immunofluorescence analysis was performed as previously described [25]. Resident leukocytes, infiltrating leukocytes, adhesion molecules (ICAM-1 and VCAM-1), blood vessels and IgG antibody deposition in the islets were detected with the following monoclonal antibodies: anti-CD11c Alexa Fluor® 488 (clone N418), anti-CD19 phycoerythrin (PE) (clone 6D5), and anti-CD4 PE (clone RM4–5) from BioLegend, Inc., San Diego, CA, anti-CD8α PE (clone 53-6.7; BD Biosciences, San Jose, CA), anti-CD31 Alexa Fluor® 647 (clone 2B8, kindly provided by Dr. Steven Bogen, Boston University School of Medicine, Boston, MA), and anti-mouse IgG Alexa Fluor® 488 (Invitrogen, Carlsbad, CA). Biotinylated antibodies against ICAM-1 (CD54, clone Yn1/1.7.4) and VCAM-1 (CD106, clone 429) were obtained from eBioscience. Streptavidin Alexa Fluor® 488 or 555 (Invitrogen) was used as the secondary reagent. Percentages of infiltrating leukocytes, adhesion molecules expression and IgG deposition were evaluated from 50 to 100 islets per mouse with groups of 4 to 6 mice per time point.

### Flow Cytometry

Isolated islets were dispersed in a water bath using Cell Dissociation Solution Non-enzymatic (Sigma-Aldrich) for 15 minutes at 37°C. Islets were then pipetted several times and then passed through a pre-wet 40 µm cell strainer and washed twice in Dulbecco’s minimal essential medium with 10% fetal calf serum. Dispersed islet cells were stained with labeled antibodies against CD45 FITC (clone 30-F11), CD4 PE (clone RM4-5), CD19 PE (clone 6D5) and CD11c APC (clone N418) from BioLegend, Inc. CD8β APC (clone eBioH35-17.2) and Foxp3 (clone FJK-16s) were obtained from eBioscience. Foxp3 staining was performed following the manufacture’s protocol. Flow cytometry analysis was performed on a FACSCalibur (BD Biosciences) and data was analyzed using FlowJo software (Tree Star).

### RNA Isolation

For islets of Langerhans (∼100), Total RNA was isolated using the Ambion RNAqueous-Micro Kit (Life Technologies, Carlsbad, CA, USA) following the manufacturer’s instructions. For spleen and lymph nodes, RNA was isolated using Trizol (Life Technologies) following the total tissue RNA protocol. RNA was quantified by OD260 using Nanodrop (Thermo Fisher Scientific, Wilmington, DE, USA). For microarray analysis, RNA integrity and quantification was further validated using a Bioanalyzer (Agilent Technologies, Santa Clara, CA, USA).

### Microarray Analysis

RNA (50 ng) was amplified using NuGEN PicoSL WTA System or NuGEN PicoPure (NuGEN, San Carlos, CA, USA) following the manufacturer’s instructions. Amplified RNA (100 ng) was labeled using Affymetrix GeneChip Whole Transcript Sense Target Labeling Assay following the manufacturer’s instructions (Affymetrix, Santa Clara, CA, USA). Labeled RNA was hybridized to Mouse Gene 1.0 ST microarrays using a GeneChip Fluidics Station 450 (Affymetrix). Microarrays were scanned using a GeneChip Scanner 3000 7G (Affymetrix). All GeneChip processing steps were performed by the Laboratory for Clinical Genomics at Washington University School of Medicine.

Initial quality control analysis of scanned microarray files was performed using Expression Console software (Affymetrix). Array data (.cel files) was imported into Arraystar 5 software (DNAstar, Madison, WI, USA) and then normalized using the robust multi-array analysis method with quantile normalization [55]. Normalized data was exported as normalized linear signal intensity and then batch effect correction was performed using the Combat module of GenePattern [16], [56]. The batches were as follows: 1) NOD.Rag^−/−^2 wk and 6 wk, NOD 2 wk, B6.g7 6 wk, C57BL/6J 6 wk; 2) NOD 4 wk, 6 wk (arrays 4–6), and newly diabetic, 3) NOD 6 wk (arrays 1–3), 8 wk, 12 wk, and 18 wk. All raw data files (CEL) are publicly available in the Gene Expression Omnibus as accession number GSE41203.

Normalized and batch corrected data was used for all subsequent analyses.

Spearman’s Rank Correlation was calculated using GENE-E Software (Broad Institute http://www.broadinstitute.org/cancer/software/GENE-E/). Principal component analysis was performed using Population PCA software (CBDM Laboratory, Harvard University, http://cbdm.hms.harvard.edu/LabMembersPges/SD.html). For all pairwise tests contained herein, we set our threshold at two-fold change with a 99% confidence interval by moderated t test with Benjamini-Hochberg False Discovery Rate analysis [57]. Hierarchical clustering analysis and heat map plot generation was performed using Arraystar 5. Gene Ontology analysis was performed using GeneCoDis 3.0 (http://genecodis.cnb.csic.es/analysis/; [22]). Promoter scanning analysis was performed using Pscan (http://159.149.109.9/pscan/; [23]) with the −950+50 settings and Jaspar mouse database. Batch name conversion was performed using the MGI Batch Query (http://www.informatics.jax.org/batch/?page=batchQF). For selecting genes specific to particular cell types we used the Immgen Modules and Regulators. The following modules were used: myeloid cells (macrophage, DC, granulocytes): coarse modules 25, 26, 48, 58, and 74, T cells: coarse module 146, B cells: coarse module 33, and NK cells: coarse module 19. Venn diagrams were generated using Arraystar 5. Pearson’s Correlation analysis was performed using Arraystar 5. Bar graphs and statistical analysis was performed using Graphpad Prism 5.0 (Graphpad Software, Inc., La Jolla, CA, USA). Hypergeometric p values were calculated using the dhyper function of R. All figures were laid out using Adobe Illustrator CS5.

### Real Time PCR

Complementary DNA was made from total RNA using TaqMan Reverse Transcription Reagents (Life Technologies) following the random hexamer protocol. Primers for quantitative RT-PCR for *Cxcl9*, *Stat1*, *Gbp2*, *Iigp1*, *Rtp4*, *Gpr18*, *Oasl2*, and *Tgtp1* were designed using Primer Bank (http://pga.mgh. harvard.edu/primerbank/; [58]. TaqMan primers and probes for amplifying and detecting *Ifng* and *Ifnb* were obtained from Life Technologies. Primers and probe for detecting all the *Ifna* transcripts were made based on previous work [59]. SYBR green PCR was performed using Fast SYBR Green PCR Master Mix (Life Technologies) following the manufacturer’s instructions. TaqMan PCR was performed using TaqMan Fast Universal PCR Master Mix (Life Technologies). All qPCRs were performed on a StepOnePlus Real-Time PCR system running StepOne Software. Quality control and relative expression quantification for qPCR was performed by the StepOne software.

### Network Analysis

The R package Bionet was used to identify subnetworks significantly enriched in genes differentially expressed at any given time point of diabetes development [60]. For each time point we scanned a range of false discovery rates and chose a subnetwork of 20–30 genes in size (p<0.05 by FDR).

Cell type enrichment scores were pre-computed enrichment scores for all genes based on mouse Immgen data. This included data for 193 immune cell types/subtypes and then applied a hypergeometric test to look for overrepresented set of genes. For each time point we chose a corresponding test set that consisted of the top 100 differentially expressed genes (all with p values <0.05). Equal numbers of genes were chosen for each time-point such that we could use a union of all test sets as background in hypergeometric test (Table S2).

## Supporting Information

Figure S1 Summarization of microarrays data throughout analysis. (**A**) Summarization of all normalized and curated genes in our dataset. (**B**) Summarization and analysis of all genes downregulated between 2 and 6 wks in NOD and NOD.*Rag^−/−^* mice. The Venn diagram shows all genes downregulated at least 2 fold at 99% C.I. following F.D.R. analysis. Genes downregulated in NOD are in red, genes downregulated in NOD.*Rag^−/−^* are in green. Shared genes are in yellow. The shared group of genes were analyzed by transcription factor biding site enrichment (Pscan) or Gene Ontology enrichment (GeneCoDis). Bar graphs show the corrected p value for each type of analysis. The heat map shows the hierarchically clustered genes shared between NOD and NOD.*Rag^−/−^* (Euclidean distance). Scale is in log_2_ fold change. (**C**) Summarization of the top 1% of variance amongst our dataset as determined by principal component analysis. For all summarization plots, we used Spearman’s rank correlation. Scales indicate the range.
(TIF)

Figure S2 Identification of genes positively correlated with diabetogenesis. (**A**) k-means clustering analysis of 21759 normalized and curated genes in our dataset. K-means was performed using Pearson’s correlation and 50 bin size at 100 iterations. The line graphs in red represent 888 genes that had a positive correlation throughout the time course of NOD diabetes but were not upregulated from 2 wk to 6 wk in NOD.*Rag^−/−^*. These 888 genes were plotted in the heat map to the right using hierarchical clustering (Euclidean distance). (**B**) Summarization of the 888 genes identified by Pearson’s correlation. Spearman’s rank correlation was used to generate the plot. Scale represents the range. (**C**) Venn diagrams showing the concordance of genes identified by Pearson’s correlation compared to pairwise statistical analysis (top) or principal component analysis (bottom).
(TIF)

Figure S3 Pearson’s correlation and ANOVA analysis demonstrate 5 main patterns of immune gene upregulation during diabetogenesis. (Left Column) Heat maps of hierarchically clustered genes identified by Pearson’s correlation and ANOVA analysis. (Right Column) Line graphs of normalized gene expression throughout diabetogenesis. Black line represents the normalized mean of expression for the cluster. The genes included in both columns were identical. We tested these 5 major gene sets that comprise 683 genes with significant changes across diabetes development by ANOVA. Each cluster can be associated with infiltration at distinct time points. Strikingly, three clusters were strongly enriched in specific immune cell types: first, “4 wk infiltration” cluster had overwhelming enrichment of macrophages and dendritic cell types (i.e. myeloid cells). Second was the cluster of genes that appeared at 8 wks and continued to grow on. This cluster had enrichment in T-cells, B-cells, NK cells and DC types. Finally, genes most strongly upregulated in newly diabetic NOD mice, where extraordinarily enriched in various cytotoxic T-cell types.
(TIF)

Figure S4 Expression levels of housekeeping, pancreas specific genes, and *Ptprc*. Normalized expression of eight genes from pancreatic microarrays. Bars represent the log_2_ transformed mean+/−S.D. for 3–6 biological replicates per group.
(TIF)

Figure S5 Data modeling of transcriptional networks**.** The analysis was performed as in Figure 6, except the (**A**) 4 wks, (**B**) 12 wks and (**C**) newly diabetic networks are shown.
(TIF)

Table S1 List of genes identified using Pearson’s correlation and pairwise statistical analysis**.**
(XLSX)

Table S2 Leukocyte clusters used in the analysis.
(XLSX)
