# Correction: Defining the Transcriptional and Cellular Landscape of Type 1 Diabetes in the NOD Mouse

**DOI:** 10.1371/annotation/f277b29e-361b-4e56-b55b-612ebaca0432

**Published:** 2014-01-13

**Authors:** Javier A. Carrero, Boris Calderon, Fadi Towfic, Maxim N. Artyomov, Emil R. Unanue

In Figure 5B the panel for "Iigp1" has been duplicated from Figure 5A. The corrected panel 5B "Iigp1" can be viewed here: http://plosone.org/corrections/pone.0059701.002.cn.tif

