# Supplementary figures and images for: Defining the Transcriptional and Cellular Landscape of Type 1 Diabetes in the NOD Mouse
Source: PLoS One. 2013 Mar 26;8(3):e59701. doi: 10.1371/journal.pone.0059701 (PMC3608568; doi:10.1371/journal.pone.0059701)

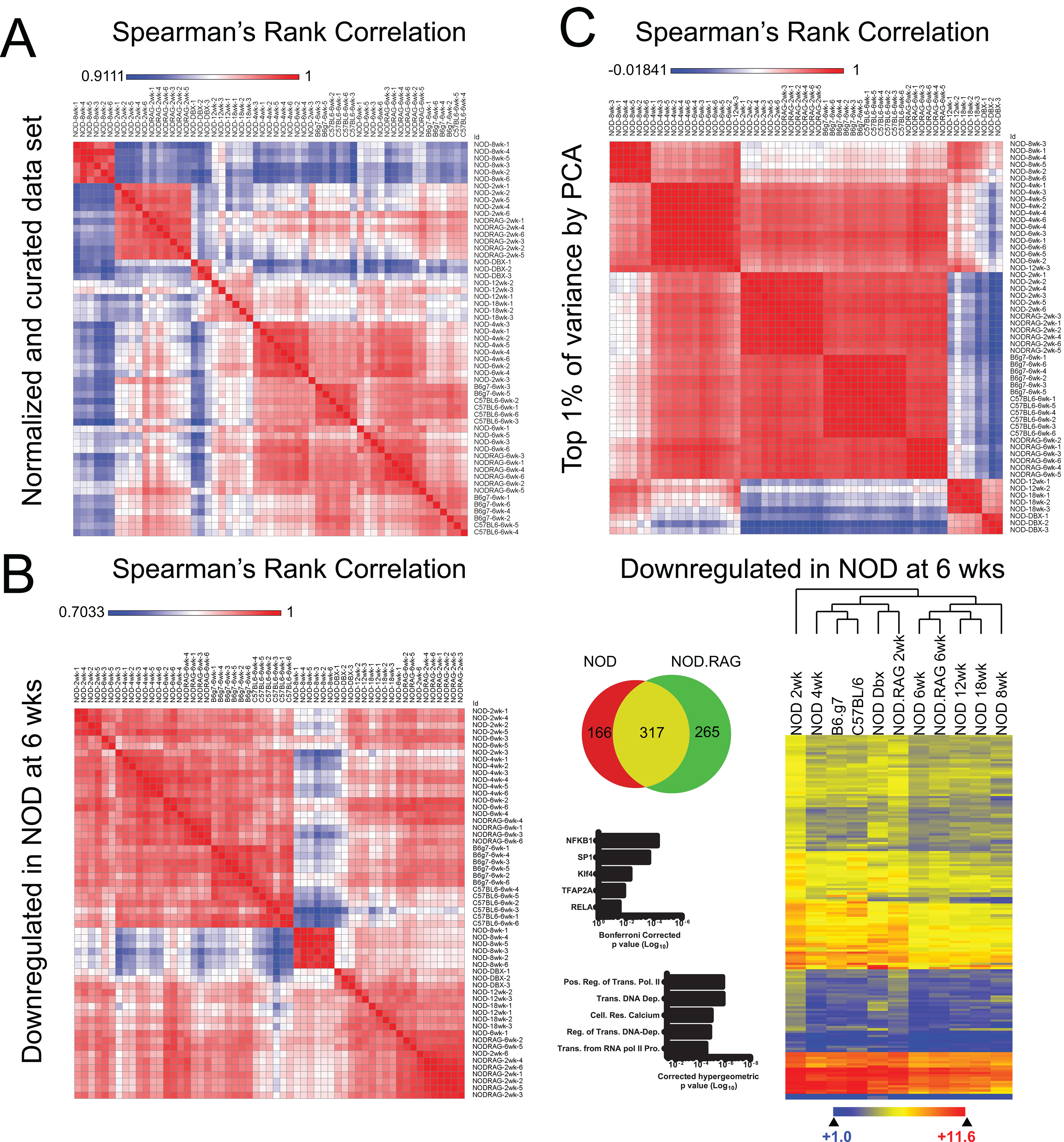

Supplement: Figure S1 — Summarization of microarrays data throughout analysis. (A) Summarization of all normalized and curated genes in our dataset. (B) Summarization and analysis of all genes downregulated between 2 and 6 wks in NOD and NOD.Rag−/− mice. The Venn diagram shows all genes downregulated at least 2 fold at 99% C.I. following F.D.R. analysis. Genes downregulated in NOD are in red, genes downregulated in NOD.Rag−/− are in green. Shared genes are in yellow. The shared group of genes were analyzed by transcription factor biding site enrichment (Pscan) or Gene Ontology enrichment (GeneCoDis). Bar graphs show the corrected p value for each type of analysis. The heat map shows the hierarchically clustered genes shared between NOD and NOD.Rag−/− (Euclidean distance). Scale is in log2 fold change. (C) Summarization of the top 1% of variance amongst our dataset as determined by principal component analysis. For all summarization plots, we used Spearman’s rank correlation. Scales indicate the range. (TIF) [file pone.0059701.s001.tif]

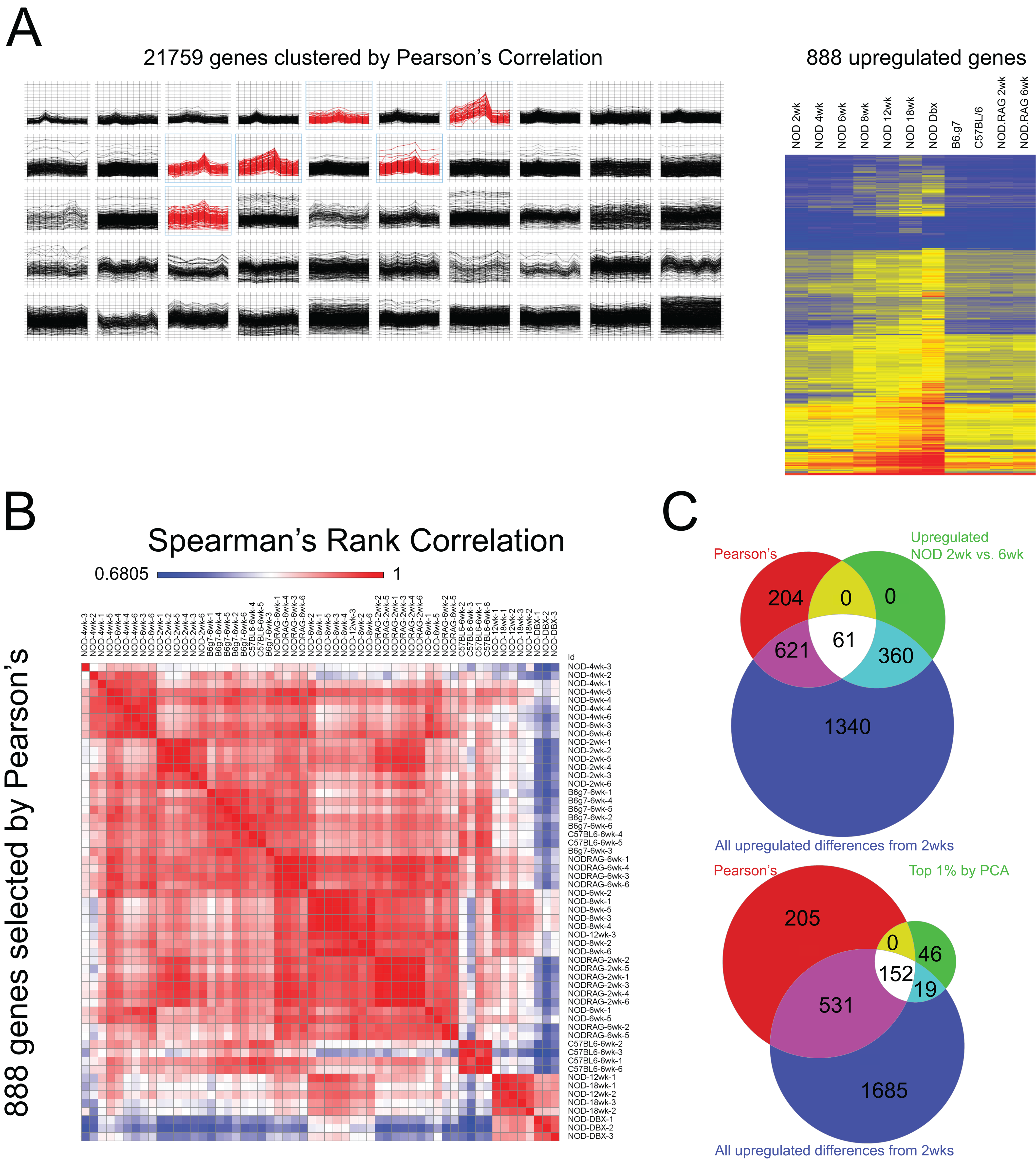

Supplement: Figure S2 — Identification of genes positively correlated with diabetogenesis. (A) k-means clustering analysis of 21759 normalized and curated genes in our dataset. K-means was performed using Pearson’s correlation and 50 bin size at 100 iterations. The line graphs in red represent 888 genes that had a positive correlation throughout the time course of NOD diabetes but were not upregulated from 2 wk to 6 wk in NOD.Rag−/−. These 888 genes were plotted in the heat map to the right using hierarchical clustering (Euclidean distance). (B) Summarization of the 888 genes identified by Pearson’s correlation. Spearman’s rank correlation was used to generate the plot. Scale represents the range. (C) Venn diagrams showing the concordance of genes identified by Pearson’s correlation compared to pairwise statistical analysis (top) or principal component analysis (bottom). (TIF) [file pone.0059701.s002.tif]

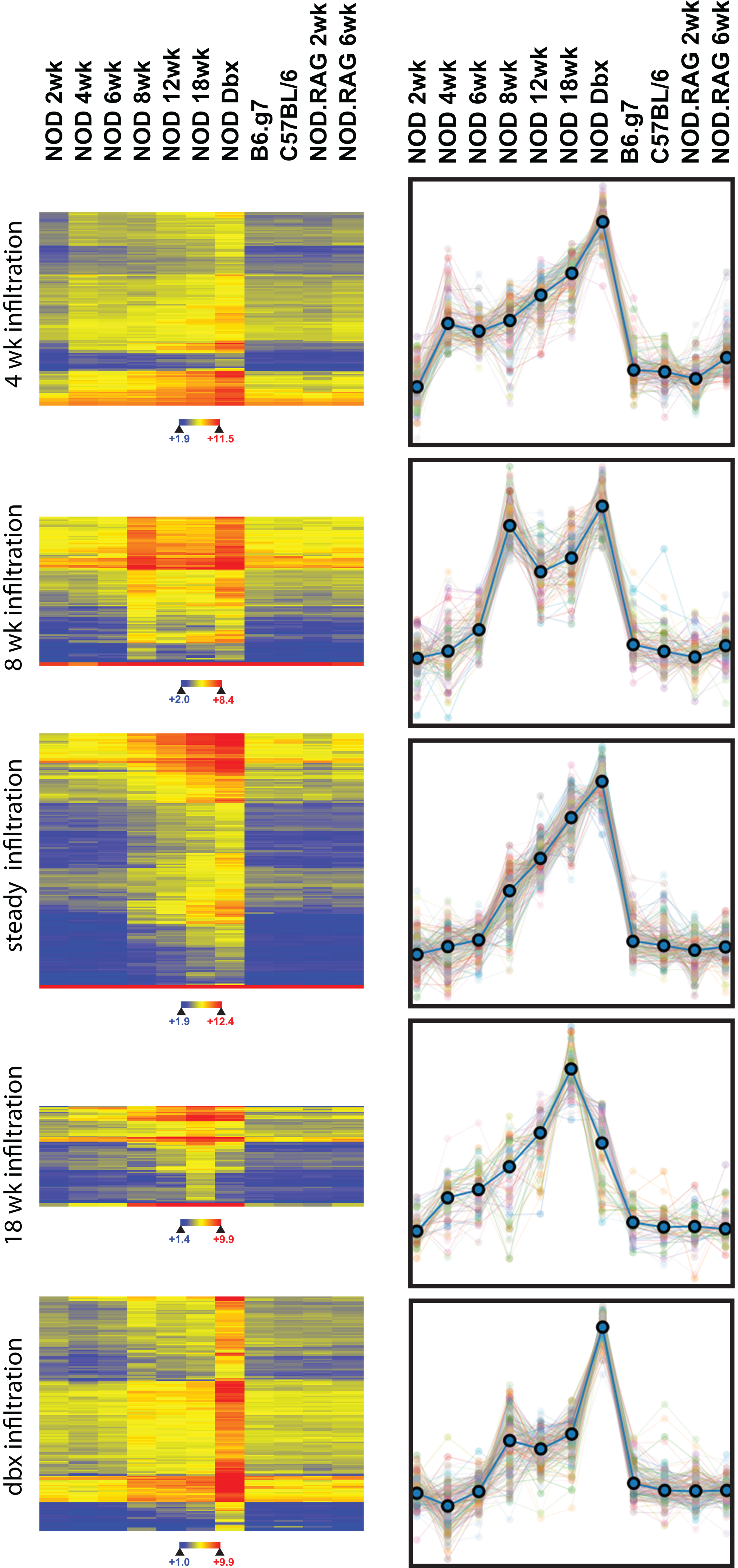

Supplement: Figure S3 — Pearson’s correlation and ANOVA analysis demonstrate 5 main patterns of immune gene upregulation during diabetogenesis. (Left Column) Heat maps of hierarchically clustered genes identified by Pearson’s correlation and ANOVA analysis. (Right Column) Line graphs of normalized gene expression throughout diabetogenesis. Black line represents the normalized mean of expression for the cluster. The genes included in both columns were identical. We tested these 5 major gene sets that comprise 683 genes with significant changes across diabetes development by ANOVA. Each cluster can be associated with infiltration at distinct time points. Strikingly, three clusters were strongly enriched in specific immune cell types: first, “4 wk infiltration” cluster had overwhelming enrichment of macrophages and dendritic cell types (i.e. myeloid cells). Second was the cluster of genes that appeared at 8 wks and continued to grow on. This cluster had enrichment in T-cells, B-cells, NK cells and DC types. Finally, genes most strongly upregulated in newly diabetic NOD mice, where extraordinarily enriched in various cytotoxic T-cell types. (TIF) [file pone.0059701.s003.tif]

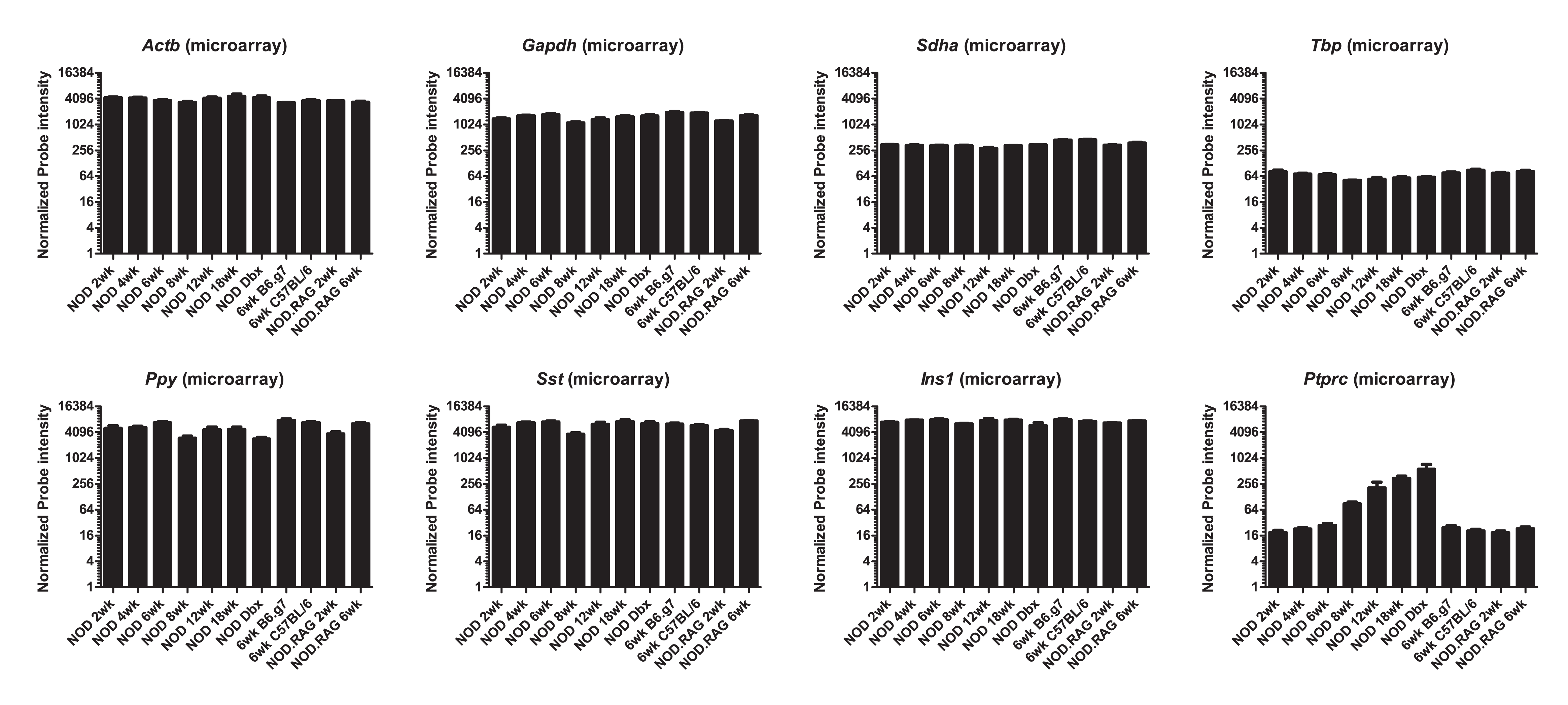

Supplement: Figure S4 — Expression levels of housekeeping, pancreas specific genes, and Ptprc. Normalized expression of eight genes from pancreatic microarrays. Bars represent the log2 transformed mean+/−S.D. for 3–6 biological replicates per group. (TIF) [file pone.0059701.s004.tif]

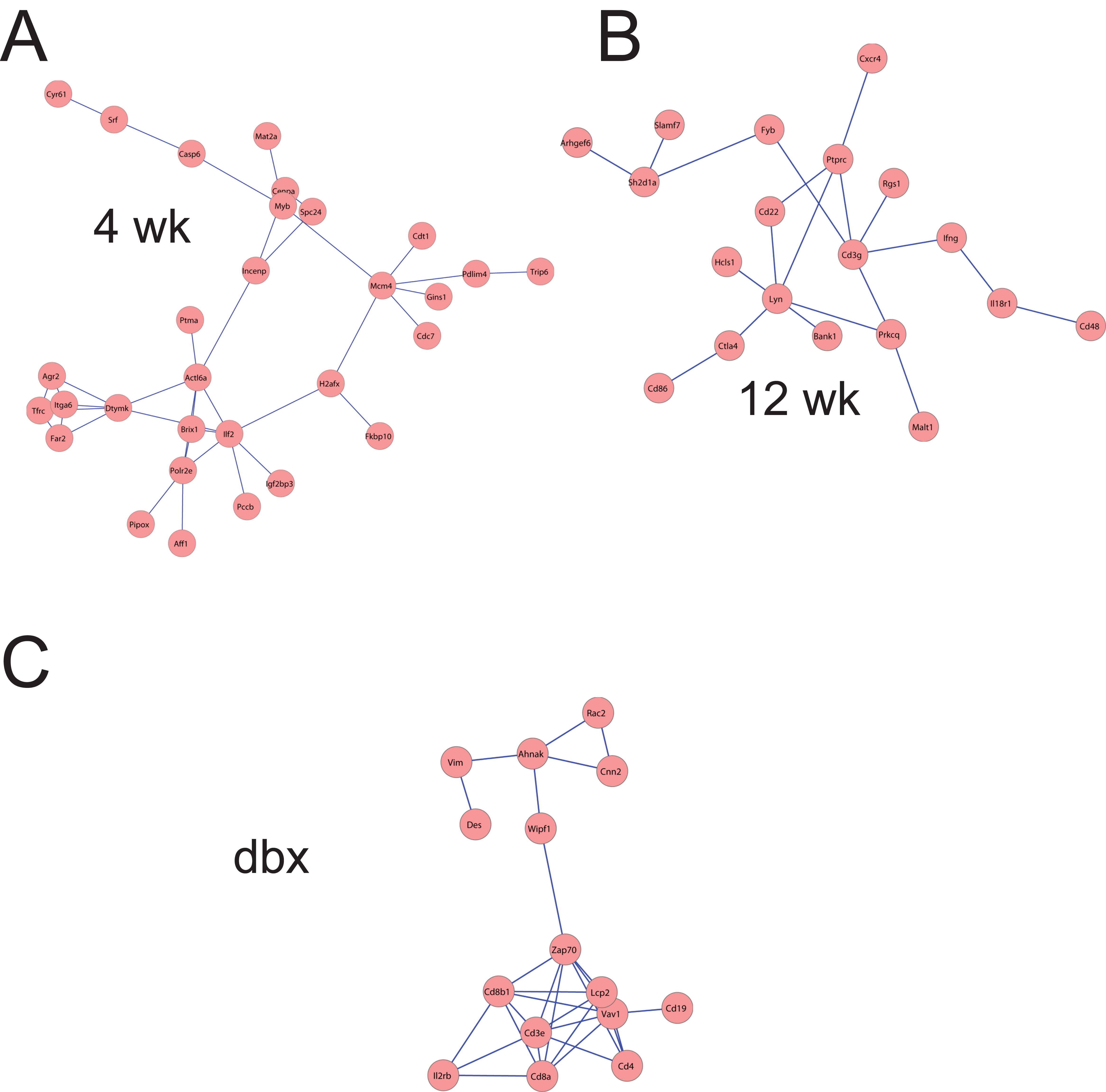

Supplement: Figure S5 — Data modeling of transcriptional networks. The analysis was performed as in Figure 6, except the (A) 4 wks, (B) 12 wks and (C) newly diabetic networks are shown. (TIF) [file pone.0059701.s005.tif]
